# Lung cancer risk among German male uranium miners: a cohort study, 1946–1998

**DOI:** 10.1038/sj.bjc.6603403

**Published:** 2006-10-17

**Authors:** B Grosche, M Kreuzer, M Kreisheimer, M Schnelzer, A Tschense

**Affiliations:** 1 Federal Office for Radiation Protection, Department ‘Radiation Protection and Health’, Ingolstaedter Landstr. 1, 85764 Oberschleissheim, Germany

**Keywords:** epidemiology, cohort study, uranium miners, lung cancer, radon

## Abstract

From 1946 to 1990 extensive uranium mining was conducted in the southern parts of the former German Democratic Republic. The overall workforce included several 100 000 individuals. A cohort of 59 001 former male employees of the Wismut Company was established, forming a large retrospective uranium miners' cohort for the time period 1946–1998. Mean duration of follow-up was 30.5 years with a total of 1 801 630 person-years. Loss to follow-up was low at 5.3%. Of the workers, 16 598 (28.1%) died during the study period. Based on 2388 lung cancer deaths, the radon-related lung cancer risk is evaluated. The excess relative risk (ERR) per working level month (WLM) was estimated as 0.21% (95% CI: 0.18–0.24). It was dependent on time since exposure and on attained age. The highest ERR/WLM was observed 15–24 years after exposure and in the youngest age group (<55 years of age). While a strong inverse exposure-rate effect was detected for high exposures, no significant association was detected at exposures below 100 WLM. Excess relative risk /WLM was not modified by duration of exposure. The results would indicate the need to re-estimate the effects of risk modifying factors in current risk models as duration of exposure did not modify the ERR/WLM and there was only a modest decline of ERR/WLM with increasing time since exposure.

From 1946 to 1990 there was extensive uranium mining in the southern parts of the former German Democratic Republic (GDR). It was conducted by the Soviet-German Incorporated Company Wismut. Some 231 000 metric tons of uranium ore were produced (Wismut, 1999) and incorporated into the former Soviet Union's nuclear programme. About 400 000 persons may have worked with the company, most of them underground or in uranium ore processing facilities (Otten and Schulz, 1998). Approximately 130 000 of the workers are known. Up to 1999, 7695 workers with radiation-induced lung cancers had been compensated (Schröder *et al*, 2002). In 2000, the annual number of newly compensated cases was still almost 200 although with a decreasing trend (Koppisch *et al*, 2004).

An increased risk of lung cancer associated with exposure to radon and its progeny among underground miners is well established (BEIR, 1999). A pooled analysis of 11 miners' cohorts revealed a linear increase of risk with increasing cumulative exposure, while the excess relative risk (ERR)/working level month (WLM) decreased constantly with increasing time since exposure and increasing attained age. Furthermore, ERR/WLM was modified by either duration of exposure or concentration. Since that study, new analyses of the North American and the Chinese cohorts have been published (Langholz *et al*, 1999; Stram *et al*, 1999; Hauptmann *et al*, 2001; Hornung, 2001; Duport, 2002; Archer *et al*, 2004; Kreisheimer, 2006), yet further follow-up was only conducted for the Czech (Tomásek, 2002; Tomásek and Zarska, 2004) and the French cohort (Rogel *et al*, 2002; Laurier *et al*, 2004). Although the evidence of a radon-related lung cancer risk among miners is large, it is based upon various heterogeneous cohorts for which the cohort-specific risk estimates vary by more than an order of magnitude. The new German cohort is as big as all the 11 cohorts put together, but less heterogeneous in various aspects: same societal and geographical background, same way of follow-up, and one system for exposure estimation.

The aim of the present analysis was to evaluate the lung cancer risk associated with radon and its progeny due to cumulative radon exposure, exposure rate, duration of exposure, time since exposure, and attained age.

## MATERIALS AND METHODS

Within the total period of uranium mining by the Wismut company, three different time periods can be distinguished which are described in detail elsewhere (Kreuzer *et al*, 2002). During the period 1946 to 1954, working conditions were characterised by dry drilling, the lack of forced ventilation, and an increasing exposure to radon. Between 1955 and 1970 the working conditions of the miners improved. Dry drilling was replaced by wet drilling and ventilation became more efficient leading to decreasing radon concentrations. After 1970 international standards for radiation protection and occupational safety were introduced, as well as individual radon measurements.

Miners' health data are stored in the Wismut Health Data Archives (Gille, 2004). These archives include paper files and histological material. The German Federation of Institutions for Statutory Accident Insurance and Prevention keeps all those data which are relevant in the course of the compensation of occupational diseases. Payrolls are kept by the successor of the old Wismut company, the Wismut GmbH. Based on information from any of these bodies the cohort could be established.

### Cohort definition

The cohort has been described previously in detail (Kreuzer *et al*, 2002; Kreuzer *et al*, 2006). In brief, the selection of cohort members was based upon three personnel files of the Wismut Company which consisted of 130 000 workers and provided personal and occupational data of sufficient detail for follow-up and for exposure estimation. In these files information on gender, year of first employment, the predominant place of work (underground, milling and processing, or surface) and the location of the mining facility (Thuringia or Saxony) was stored, whereas the corresponding detailed job histories were extracted from the original pay rolls. A stratified random sample of 64 311 workers was drawn. In order to reflect the different mining conditions at the Wismut company the sample was stratified by the date of first employment (1946–1954, 1955–1970, 1971–1989), place of work and area of mining. In order to achieve a large number of low exposed workers all miners were included whose first employment was 1971 or later. Thus the cohort does not reflect the composition of the former Wismut workforce but was established to allow risk estimates based on high, medium, and low exposures within one single cohort to be determined. As it was assumed that during the early years women also worked underground for at least some time, the sample was additionally stratified by gender. Inclusion criteria at the time of data collection were a date of first employment between 1946 and 1989 with a minimum duration of employment of 180 days.

A total of 5310 individuals (8.3%) who did not meet the inclusion criteria for this analysis were excluded from the initial cohort: all 4194 females; 799 individuals born before 1900, due to the results of a pilot study (Blettner *et al*, 1997); 260 individuals because their date of first employment was later than 31 December 1989 (i.e. they were employed in order to do work related to the closedown of the mines); and 35 individuals who were double entries in the database. A further 160 individuals were excluded due to incomplete information. Thus, the cohort consisted of 59 001 male workers.

### Information on exposure to radiation

Radiation exposure was estimated by using a job-exposure matrix (JEM), which was developed for compensation purposes by the Miners' Institution for Statutory Accident Insurance and Prevention and was first described in detail in 1998 (Lehmann *et al*, 1998). The JEM has been developed further to meet scientific purposes (Lehmann, 2004). For 1946 to 1955 radon exposure had to be determined retrospectively, since there was no dosimetric recording. Radon concentrations during this time period were estimated based on the first available radon measurements in 1955 taking into account previous working conditions in the mines and mine architecture as well as historical measurements and data gathered from the Czech and French ore mining industry. Radon gas monitoring was carried out after 1955, while measurements of radon progeny began in 1966. Based on these measurements, more than 900 different jobs and several mining facilities have been evaluated by a group of experts and were used as a basis for the JEM. The JEM provides the exposure values for radon and its progeny in WLM for each calendar year of employment between 1946 and 1989, mining facility, place of work (underground, milling and processing, or surface), and type of job (A working level (WL) is defined as 1.3 × 10^5^ MeV of potential alpha energy/l air. A WL Month equals exposure to 1 WL for 170 h. For the data analysis, the annual mean WL was calculated by dividing the annual WLM by 11). The information on jobs is available on a daily basis, including times of absence, for example, due to illness, or the number of underground shifts in a given year for employees who were not exposed otherwise.

The cumulative radon exposure for each cohort member was calculated as the sum of the annual radon exposures. A worker with 0 WLM was defined as unexposed, a worker with an exposure exceeding 0 WLM was considered as exposed. Table 1 shows the exposure characteristics for different workplaces. The largest proportion of the cohort members had worked underground. Overall, the mean exposure was 241 WLM with 332 WLM among underground workers and 235 WLM among those having worked at different exposed work places, respectively. For the other exposed workers, mean exposures were below 10 WLM. The highest cumulative exposure was 3225 WLM.

The mean age at first exposure was 24.6 with one third of all workers having been exposed for the first time below the age of 20 years. The mean duration of exposure was 11.3 years, while almost 40% of the cohort members had worked 5–14 years. Almost one third of the exposed workers experienced exposures below 10 WLM; while for 9.3 % of them exposure exceeded 1000 WLM.

### Mortality follow-up

Basically, the follow-up was conducted via local registration offices to find the most recent place of residence. If a subject was deceased, a copy of the death certificate was requested from local health authorities. For cases of death before 1989, causes of death were determined from the Wismut Pathology Archive and from District Archives.

### Analysis

Number of person-years was calculated starting 180 days after date of first employment and ending at date of loss to follow-up, date of death, or end of follow-up (31 December 1998), whichever came first.

For all analyses, a lag time of 5 years was introduced. Data analysis was conducted using Poisson regression assuming a linear relation between exposure and risk. We used the same program and the same – yet slightly modified – models that were used for the BEIR-VI-Report (BEIR, 1999), that is, EPICURE (Preston *et al*, 1993), the exposure-age-concentration (EAC) model and the exposure-age-duration (EAD) model, respectively.

In the first step, ERR per WLM was estimated based on the accumulated exposure only, stratified by age and by calendar year. In the second step, the following windows were used for the analyses: for time since exposure 5–14, 15–24, 25–34, and 35 and more years; for attained age <55, 55–64, 65–74, and 75+ years. Depending on the model, that is, EAC or EAD, the third windows were <0.5, 0.5–1.0, 1.0–3.0, 3.0–5.0, 5.0–15.0, and 15+ WL for EAC and <5, 5–14, 15–24, 25–34, and 35+ years for EAD, respectively. All parameters are given with their 95% Wald-type confidence limits as computed by EPICURE's AMFIT module.

Results from other studies indicate that the magnitude of an inverse exposure-rate effect depends on the total accumulated exposure (Lubin *et al*, 1995). To test for such an effect and for its possible exposure dependence, analyses were conducted for four groups of workers with different accumulated exposures, that is, all exposed workers and workers with a total accumulated exposure of less than 500 WLM, 200 WLM, and 100 WLM, respectively. Again, exposure was considered as a time dependent variable, while exposure-rate was calculated as the mean rate over the time period of exposure.

Finally, the lifetime attributable risk (LAR) for radon-induced lung cancer was estimated. An equation for LAR is given by Vaeth and Pierce (1990) and Kellerer *et al* (2001) 
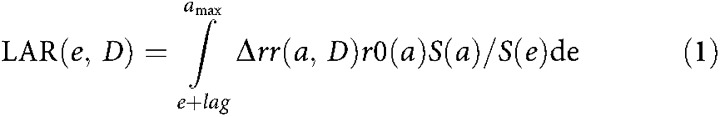
 with *r*_0_(*a*) being the spontaneous lung cancer rate, and Δ*rr*(*a*, *D*) the ERR.

The survival function, that is, the probability at birth of reaching at least age *a*, is denoted *S*(*a*). The ratio *S*(*a*)/*S*(*e*) is the conditional probability of a person alive at age *e* reaching at least age *a*. Integration in Eq. (1) starts at age of first exposure *e* plus lag time and a survival probability of 1 is assumed for this age.

Here, a survival probability of 1 is assumed after birth and a slightly modified equation was utilised with start point for integration at 0 
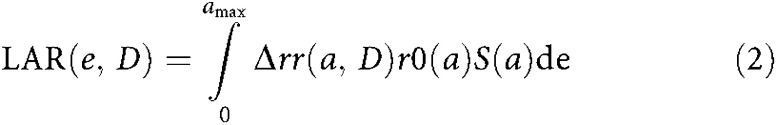


The survival probabilities *S*(*a*) were those for East German males and were taken from official statistical data.

## RESULTS

Overall, the 59 001 cohort members contributed 1 801 630 person-years with a mean duration of follow-up of 30.5 years. 3148 (5.3%) of the cohort members were lost to follow-up (1905 could not be identified, 677 moved to unknown new addresses, 427 were refugees to former Western Germany, 72 moved abroad, and for 67 the date of death was unknown). 16 598 (28.1%) of the cohort members were deceased, while for 14 646 (88.2%) of them a cause of death could be determined. Among all deaths with known cause, 2388 (14.4%) were due to lung cancer.

Of the lung cancer cases, 187 cases were unexposed and 2201 were exposed. The number of person-years was 236 560 and 1 565 070, respectively. This gave a relative risk, adjusted for age and calendar period, of 2.08 (95% confidence intervals (CI)=1.08–2.79) for the exposed. Taking into account only accumulated exposure as a time-dependent variable and stratifying for age and calendar period, the ERR/WLM was 0.21% (95% CI=0.18–0.24%).

Results using the EAC model are shown in Table 2. The ERR/WLM was highest 15–24 years after exposure, while it was significantly lower in the other three categories of time since exposure. The confidence limits for these three groups overlapped. The decline of ERR/WLM with attained age was modest. The effect of concentration (or exposure rate) was strong, and an inverse exposure-rate effect was indicated, that is, ERR/WLM increased with decreasing exposure-rate.

The ERR/WLM was not statistically significantly modified by duration of exposure when applying the EAD model (see Table 2). The estimates for the other two parameters were only slightly different from those using the EAC model. Table 2 also shows the deviance for each of the models and the likelihood ratio statistic for the improvement of the model by adding the third parameter, that is, concentration and duration, respectively. For the first one it was 45.6 (degrees of freedom (df)=5, *P*<0.001), for the latter 23.7 (df=4, *P*<0.001).

**Figure 1:**
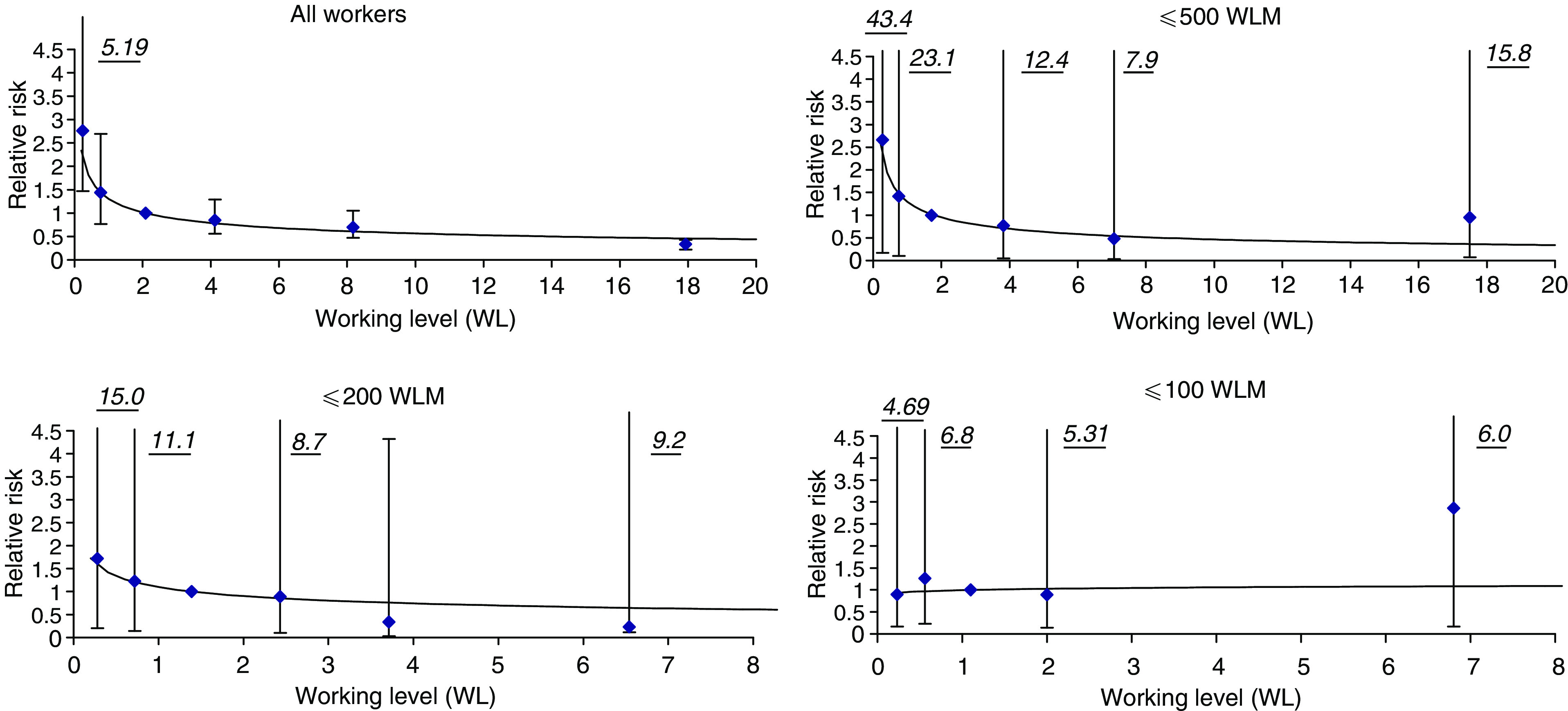
Lung cancer risk in relation to exposure rate, given in WL, for different groups of accumulated exposure (italic figures give upper limit of 95% CI). The risk in the third category was used as reference.

Figure 1 shows the results of a more detailed analysis of the exposure-rate effect. A pronounced inverse exposure-rate effect was present when using the data of all miners (Figure 1, upper left). Compared to the reference exposure-rate category (i.e. Category 3), the risks in the lowest category and in the highest category were significantly higher and lower, respectively. This effect became smaller the more the analysis was restricted to lower accumulated exposures. No inverse exposure-rate effect for exposures up to 100 WLM was detected.

As an example for estimating the lifetime attributable lung cancer risk, LAR was calculated for a man who worked from age 20–39 years with an accumulated exposure of 500 WLM and a uniform exposure over time, that is, 25 WLM per year. The background risk using the Wismut model was estimated to be 7.7% and the predicted additional lifetime risk to be 10.0%.

## DISCUSSION

This is the first lung cancer risk analysis of the German uranium miners' cohort, the Wismut cohort. We have analysed the data according to the methods used for the joint analysis of 11 radon exposed miners' cohorts (BEIR, 1999). The Wismut cohort is comparable to these jointly analysed 11 cohorts in size and in number of lung cancer cases, whereas the duration of follow-up is longer in the Wismut cohort. Moreover, the Wismut cohort is less heterogeneous than the group of 11 cohorts in several aspects: all cohort members have the same geographical and societal background, follow-up was conducted in the same manner, exposure estimates were based on the same JEM for all cohort members, and causes of deaths were coded by one professional coder using ICD-10 (WHO, 1992).

We found a statistically significant trend of risk for lung cancer with increasing exposure. The overall estimated ERR was 0.21%/WLM (95% CI=0.18–0.24) and thus lower than the one given in the BEIR VI report (BEIR, 1999), that is, 0.76%/WLM. The CI of our findings did not include the value of 0.76, but no confidence limits were given in the BEIR VI report. As mentioned in this report, the estimates based on the different single cohorts range from 0.09% (France) to 4.76% (Radium Hill). Our estimate lay within this range. The differences among the various cohorts might reflect their heterogeneity. More recent results for the Czech and the French cohorts were based on further follow-ups, and ERR/WLM was estimated as being 1.8 % (95% CI=1.3–2.5) (Tomásek and Zarska, 2004) and 0.6% (95% CI=0.1–1.2) (Laurier *et al*, 2004), respectively. While the estimate for the Czech cohort is significantly different from ours, the CIs for the estimates of the German and the French cohorts overlap.

In our data, the effect of exposure to radon was modified by time since exposure and attained age. While ERR/WLM was modified by exposure-rate, no risk modifying effect of duration of exposure was found. Although the improvement of the model by adding duration of exposure was statistically significant, there was no statistically significant difference between the estimates for the various windows of duration. Thus, our ‘preferred model’ is the EAC model.

To be comparable with BEIR VI, we reanalysed the data using the same three time windows 5–14, 15–24, 25+ years for time since exposure. Table 3 summarises the modifying effects of the parameters included in the EAC model for our data set and for the 11 cohorts. In our cohort the highest risk was observed 15–24 years after exposure, whereas it was highest 5–14 years after exposure based on the 11 cohorts. Our finding of the highest values for ERR/WLM 15–24 years after exposure is consistent with what has been shown for American uranium miners. For the Colorado miners, the highest risk was estimated for 14 years since exposure (Hauptmann *et al*, 2001) and 17 years since exposure (Kreisheimer, 2006), based on different statistical approaches. When taking into account the American Underground Uranium Miners (UGUM) data set, the radon-related mean latency period was estimated as 25 years for former and nonsmokers and 19 years for cigarette smokers, respectively (Archer *et al*, 2004).

The effect of attained age was similar to that reported in BEIR VI, showing highest risks in the youngest age group (below 55 years of age). Yet, the decrease of risk was less pronounced in the Wismut cohort than it was in the 11 cohorts.

The inverse effect of exposure rate was strong for the entire cohort. Our findings of a risk modifying effect of the exposure rate, that is, concentration of potential alpha energy in air, are consistent with that in the BEIR VI report. Early reports on such an effect were given for lung cancer rates among Colorado miners (Hornung and Meinhardt, 1987) and for Czech miners (Sevc *et al*, 1988). For the latter, the result has been reproduced (Tomásek *et al*, 1994).

We found that the inverse exposure-rate effect diminished with decreasing cumulative exposure. It can still be seen below 500 WLM, but differences in relative risk did not reach statistical significance (Figure 1). The same accounts for exposures below 200 WLM. Below 100 WLM, there was clearly no such effect. The fact that the inverse exposure-rate effect can only be seen at high doses is in accordance with results from other studies. When pooling original data from 11 cohort studies, no such an effect was seen below 50 WLM (Lubin *et al*, 1995), and an analysis of the French cohort among miners with low exposures and low exposure rates showed no evidence for this effect either (Rogel *et al*, 2002). In contrast to our findings, a recent case–control study among former Wismut uranium miners where subjects experienced high exposures showed no such inverse exposure-rate effect (Brüske-Hohlfeld *et al*, 2006). The case–control study included incident lung cancer cases from the Wismut work force diagnosed between 1991 and 1999 who predominately started work in the early years and experienced a long survival time. Thus, the study's results may be biased by a healthy survivor effect. There is no such bias in our cohort study.

The inverse dose-rate effect – which is called exposure-rate effect throughout this paper since we used information on exposures as a surrogate for doses – can be explained on a microdosimetric basis (Rossi and Kellerer, 1986; Brenner and Hall, 1990). During the cell cycle, one or several periods might show a much higher sensitivity to irradiation than the others. Protracted exposure to *α*-emitters over a longer time leads to a larger proportion of these sensitive cells compared to the same exposure during a shorter time. It has further been suggested that reduction of dose-rate may allow for proliferation of cells being initiated by radiation earlier during exposure (Moolgavkar *et al*, 1990), and it was pointed out that the inverse dose-rate effect depends on total dose (Brenner, 1994), which is in accordance with our findings. Another suggestion is that this effect could be explained as a bystander effect (Brenner and Sachs, 2003), which has been successfully modelled for the data set of the 11 cohorts (Little, 2004).

The risk modifying effect of duration of exposure, as described in the BEIR-VI-Report, could not be confirmed in our study. For the entire cohort, the mean totally accumulated exposure correlated with duration of exposure, but not among the lung cancer cases (see Table 4). This result was the same for both the exposed cohort members and the entire cohort (data not shown).

**Figure 2:**
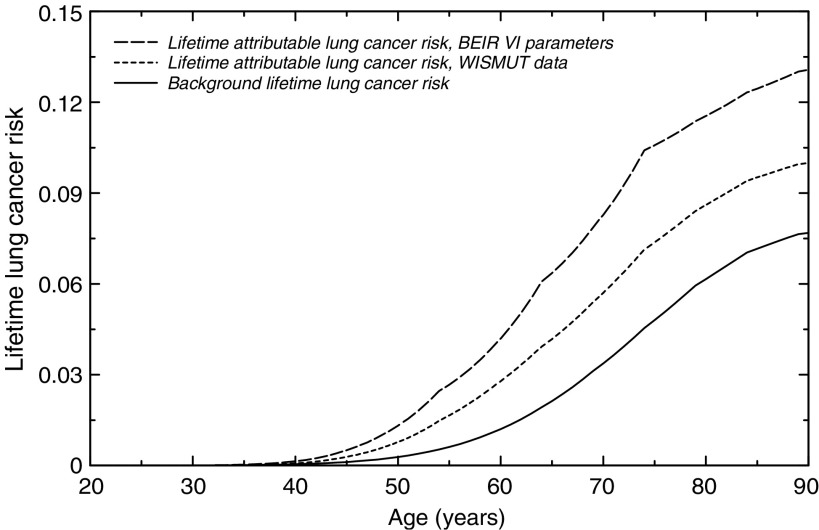
Lifetime attributable lung cancer risk for an assumed exposure of 20 years between ages 20 and 39 years and a resulting total accumulated exposure of 500 WLM (annual exposure: 25 WLM, exposure rate: 2.273 WL), using BEIR VI and WISMUT parameter estimates. Also given is the background lifetime lung cancer risk, estimated from the WISMUT data.

To estimate the implication of the differences between the Wismut model and the BEIR-VI-model on risk projection, we compared the LAR for the same man as mentioned above. The background risk from the Wismut model was estimated to be 7.7% and LAR to be 10.0%. Based on the BEIR VI model, LAR is estimated to be 13.1%. This indicates that both models give comparable results for risk projection (see Figure 2). It is noteworthy that the predicted background rate of 7.7% based on the Wismut model is close to the lung cancer rate among the male population of the former GDR of 7.9%.

The case–control study among incident lung cancer cases from the former Wismut work force found an increase of the ERR/WLM for 45 or more years since exposure (Brüske-Hohlfeld *et al*, 2006). To test this effect, we reanalysed our data set using five time windows. The strong and statistically significant increase of ERR/WLM for 45 or more years since exposure was not repeated in our study (see Table 5).

A potential limitation of the study is the 11.8 % of missing information on the cause of death. A review of the data, however, suggested that the percentage of missing lung cancer cases was well below this figure. A large proportion of the miners' information on causes of death was available from the pathology archive of the Wismut Company, and those who died from cancer and particularly lung cancer had a higher chance of *post-mortem* autopsy. 58% (1385 cases) of all lung cancer deaths were verified by autopsy. Among these, information for 447 deaths was based only on autopsy records from the pathology archive. Taking into account only the remainder of 1941, we estimated the number of missing lung cancer cases by an imputation method (Rittgen and Becker, 2000). According to this analysis 452 lung cancer cases would have been missed if the analysis was based on official records only. This proportion is similar to the number of cases for which information was only available from the archive. Both the fact that a large proportion of the lung cancer cases were based on records from the pathology archive and the result of the imputation method indicate that we have not missed a substantial number of lung cancer cases.

Several validity checks were completed for vital status ascertainment. We first compared the data on vital status as of the pathology archive (all deceased) with the data received from the local registries (*n*=2382). About 1% of the deceased cohort members from the pathology archive had falsely been specified as ‘alive’ by the local registries. A second strategy compared the data on vital status from all deceased persons according to the records of the Wismut company (excluding those with additional information from the pathology archive) with the data received from the local registries (*n*=1905). Only 2% had been wrongly classified by either the Wismut Company or by the local registries. In all cases this was due to erroneous spelling or falsely identified persons (e.g. same name but different year of birth). We estimate that for about 1% of the cohort the vital status may be wrong. The effect on risk estimates is supposed to be small.

The most important risk factor for lung cancer is smoking, even among uranium miners. In general, smoking information for cohort members is only available since 1971, when standardised and well-recorded medical check-ups were introduced. Subsequently, such information is available for 38% of the cohort members and was not used within the current analysis. However, the authors of the above-mentioned case–control study report a slight inverse correlation between radon exposure and smoking (Brüske-Hohlfeld *et al*, 2006). The ERR/WLM increased when the analysis was adjusted for smoking. Thus, if there should be an influence of smoking on the radon-related lung cancer risk within the cohort, the effect would be small and if at all bias the estimate towards the null.

**Figure 3:**
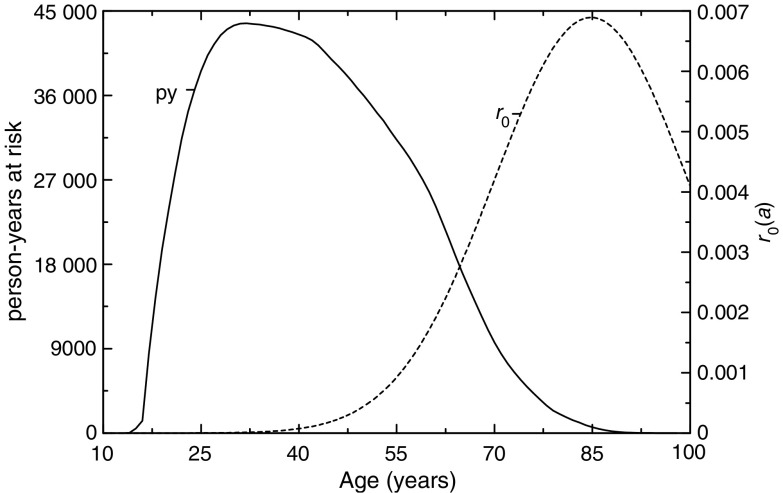
Person-years (PY; solid line) and baseline risk (*r*_0_; dotted line) as estimated from the cohort data by age.

A difference between the 11 cohorts and the Wismut cohort was the number of person-years. While the joint analyses covered 888 906 person-years, the respective number for the Wismut cohort was 1 801 630. Although the number of person-years in the latter study was about twice that of the first one, the number of lung cancer cases was similar (2674 and 2388, respectively). This is not due to an under ascertainment of lung cancer cases, but due to the fact that the Wismut cohort is rather young. As can be seen from Figure 3, the majority of person-years was in an age range where there are only few cases to be expected.

The strength of our study is its size and homogeneity, which allowed the verification of current knowledge on lung cancer risk from underground occupational exposure to radon and its progeny based on an independent data set. The percentage of loss to follow-up was small, particularly taking into account the late start of the study. The exposure estimates were based on a very detailed JEM. The cohort is relatively young and includes a large number of individuals with low exposures. So in the future it will be possible to estimate the lung cancer risk due to low levels of exposure, which are close to what is observed in a normal housing environment (Darby *et al*, 2005).

Limitations of the study include uncertainties in the estimation of exposure, particularly in the early years. We analysed three subgroups of the cohort according to the above-mentioned time periods of quality of exposure assessment (<1955, 1955–1964, 1965+). We used the EAC model. For those who were first employed before 1955, 1927 lung cancer cases occurred within 895 453 person-years. The mean exposure was 527 WLM. Results were similar to those for the entire cohort. For those having started work 1955–1964 (402 cases, 466 049 person-years, mean exposure 104 WLM) there was only a weak effect of exposure-rate. This was compatible with our overall observation of no inverse exposure-rate effect at low accumulated exposures. No conclusion can be drawn for those having started work after 1964 (59 cases, 440 124 person-years, mean exposure: 11 WLM). It was impossible to evaluate whether observed differences between the three subgroups were due to quality of exposure assessment or due to differences in accumulated exposures.

Previous studies have shown that arsenic and dust are important confounding factors when evaluating the radon-related lung cancer risk (Chen and Chen, 2002; Tapio and Grosche, 2006). Both dust and arsenic play a role in the working environment of the German uranium miners. The current analysis did not consider these exposures.

Overall, this analysis of the Wismut cohort data was based on the models described in the BEIR VI report (BEIR, 1999). It gave an overall ERR for lung cancer of 0.21 %/WLM, which was modified by time since exposure, attained age, and exposure rate. An inverse exposure-rate effect was observed at exposures exceeding 100 WLM, and it increased with increasing accumulated exposure. The derived estimates were based on a large and homogeneous cohort with a broad dose range. The results give cause to re-estimate the effects of risk modifying factors in current risk models.

## Figures and Tables

**Table 1:** Cumulative exposures to radon progeny (in WLM) among the Wismut miners cohort members

		**WLM**		
**Place of work**	**Cohort members**	**Mean**	**Min**	**Max**
All exposed	50 757	280.2	>0	3 224.5
Underground^a^	39 726	332.1	>0	3 224.5
Processing/milling^b^	4451	8.1	0.01	126.9
Open pit mining^c^	1277	3.4	>0	34.6
Surface^d^	1093	1.8	0.01	28.0
Mixed exposure^e^	4210	235.2	>0	2790.6
Not exposed	8244	0	0	0
All	59 001	241.1	0	3224.5

WLM=working level month.

a Underground only.

b Processing/milling only.

c Open pit mining only.

d Surface only, other than open pit mining or milling and processing.

e Exposed at different work places.

**Table 2:** Estimates of exposure-related lung cancer risk using time since exposure and attained age categories as well as exposure rate (concentration) and duration of exposure categories, respectively

**Model**	**Exposure-age-concentration**	**Exposure-age-duration**	
Observed lung cancer cases	2388	2388	
Estimated excess cases	1,221.7 (51.2%)	1,067.7 (44.7%)	
Deviance	13 221.97	13 244.23	
ERR/WLM (%)	0.24 (0.13–0.34)^a^	0.25 (0.11–0.39)	
			
*Time since exposure (years)*			
5–14	0.69 (0.45–0.93)	0.67 (0,40–0.93)	
15–24	1.0	1.0	
25–34	0.58 (0.44–0.72)	0.65 (0.48–0.83)	
35+	0.42 (0.31–0.53)	0.50 (0.36–0.65)	
			
*Attained age (years)*			
<55	1.0	1.0	
55–64	0.80 (0.64–1.0)	0.78 (0.62–0.98)	
65–74	0.69 (0.52–0.92)	0.64 (0.48–0.86)	
75+	0.55 (0.33–0.91)	0.50 (0.30–0.84)	
			
**WL**	**Years**	**Exposure rate (WL)**	**Duration of exposure (years)**
<0.5	<5	8.10 (4.27–15.2)	1.0
0.5–1.0	5–14	4.30 (2.30–8.02)	1.38 (0.63–3.06)
1.0–3.0	15–24	2.96 (1.92–4.57)	2.04 (0.92–4.0)
3.0–5.0	25–34	2.50 (1.64–3.79)	1.58 (0.70–3.53)
5.0–15.0	35+	2.08 (1.38–3.12)	1.16 (0.46–2.93)
15.0+		1.0	
LR statistic		45.56 (df=5, *P*<0.001)	23.66 (df=4, *P*<0.001)

df, degrees of freedom; ERR=excess relative risk; WL=working level; WLM=working level month.

a 95% Confidence intervals.

*P*=*P*-value.

**Table 3:** Exposure-age-concentration model: comparison of BEIR VI parameters (BEIR, 1999) with parameters estimated from the WISMUT cohort

	**BEIR VI**	**Wismut cohort**
ERR/WLM (%)	0.83	0.25 (0.13–0.36)
		
*Time since exposure (years)*		
5–14	1.0	0.66 (0.44–0.89)
15–24	0.78	1.0
25+	0.51	0.50 (0.40–0.60)
		
*Attained age (years)*		
<55	1.0	1.0
55–64	0.57	0.80 (0.64–1.0)
65–74	0.29	0.66 (0.50–0.88)
75+	0.09	0.49 (0.30–0.83)
		
*Exposure rate or concentration (WL)*		
<0.5	9.09	8.19 (4.36–15.4)
0.5–1.0	4.45	4.27 (2.28–8.0)
1.0–3.0	3.36	2.97 (1.93–4.57)
3.0–5.0	2.91	2.52 (1.66–3.83)
5.0–15.0	1.55	2.08 (1.39–3.13)
15.0+	1.0	1.0

BEIR=US National Academies, Nuclear and Radiation Studies Board (formerly: Board on Radiation Effects Research), Committee on the Biological Effects of Ionising Radiation; ERR=excess relative risk; WL=working level; WLM=working level month.

**Table 4:** Parameter estimates for effect modification by duration of exposure for the Wismut cohort, mean doses by duration of exposure for all workers and for cases, given in BEIR VI categories (BEIR, 1999) for duration of exposure

		**All workers**		**Lung cancer cases**	
**Duration (years)**	**Parameter estimate**	**No. of workers**	**Mean exposure (WLM)**	**No. of cases**	**Mean exposure (WLM)**
<5	1.0	17 714	53.90	280 (1.6%)	135.74
5–14	1.35 (0.63–2.87)	20 023	243.34	735 (3.6%)	637.78
15–24	1.97 (0.92–4.22)	10 022	328.67	653 (6.5%)	864.85
25–34	1.54 (0.71–3.33)	7509	371.72	488 (6.5%)	777.93
35+	1.05 (0.43–2.55)	3733	619.19	232 (6.2%)	849.37
All		59 001	241.08	2388 (4.01%)	690.20

BEIR=US National Academies, Nuclear and Radiation Studies Board (formerly: Board on Radiation Effects Research), Committee on the Biological Effects of Ionising Radiation; WLM=working level month.

**Table 5:** Risk modifying effect of time since exposure for the Wismut miners cohort data and ERR/100WLM for a Wismut miners case–control study (Brüske-Hohlfeld *et al*, 2006)

**TSE^a^ (years)**	**Cohort study**	**Case–control study**
5–14	0.69 (0.45–0.93)^b^	1.28 (−0.62–4.15)
15–24	1.0	
25–34	0.57 (0.43–0.72)	0.20 (−0.15–0.68)
35–44	0.42 (0.30–0.53)	0.07 (0.01–0.14)
45+	0.57 (−0.072–1.21)	0.36 (0.15–0.69)

ERR=excess relative risk; WLM=working level month.

a Time since exposure.

b 95% Confidence interval.
